# LncRNA HOTAIR Contributes to Sorafenib Resistance through Suppressing miR-217 in Hepatic Carcinoma

**DOI:** 10.1155/2020/9515071

**Published:** 2020-05-09

**Authors:** Xiaofeng Tang, Weichen Zhang, Yufu Ye, Hong Li, Longyu Cheng, Min Zhang, Shusen Zheng, Jun Yu

**Affiliations:** Division of Hepatobiliary and Pancreatic Surgery, Department of Surgery, First Affiliated Hospital, School of Medicine, Zhejiang University, Hangzhou, Zhejiang 310003, China

## Abstract

**Background:**

Sorafenib is a multi-target kinase inhibitor that has been approved as a unique target drug for the treatment of advanced hepatocellular carcinoma (HCC). However, due to the frequent occurrence of drug resistance, its treatment efficacy is often limited. The aim of this study was to explore the function of HOX transcript antisense intergenic RNA (HOTAIR) for the treatment of HCC with sorafenib, and its underlying mechanism.

**Methods:**

A cell counting kit-8 (CCK-8) assay and Edu assay were used to examine the viability and proliferation of HCC cells. Quantitative real-time polymerase chain reaction (qRT-PCR) was used to detect the expression of HOTAIR and miR-217 in HCC cells. Small interfering (si) RNA was transfected to knockdown HOTAIR to explore its biological function. A Western blot and immunofluorescence were performed to detect the level of E-cadherin and Vimentin expression.

**Results:**

Sorafenib resistance was increased in HCC cells with high HOTAIR expression. Moreover, a knockdown of HOTAIR could improve the therapeutic effect of sorafenib on HCC via increasing E-cadherin and decreasing Vimentin expression. Additionally, a HOTAIR knockdown could increase the sensitivity of sorafenib for HCC treatment by up-regulating miR-217.

**Conclusions:**

Lnc HOTAIR could increase sorafenib resistance in HCC by inhibiting miR-217. Our research attempts to elucidate a more effective treatment and provides novel insight into potential clinical treatment for HCC.

## 1. Introduction

Hepatocellular carcinoma (HCC) is the fifth most common cancer globally, and the third most common cause of cancer-related death [1]. Since the initial stage of HCC is not associated with any obvious symptoms, most patients are diagnosed with advanced disease, at which stage the treatment efficacy is limited [2]. Sorafenib is a multi-target kinase inhibitor that suppresses tumor cell proliferation and angiogenesis [3, 4]. Sorafenib was approved by the FDA in 2007 as a unique target drug for advanced HCC; however, its treatment efficacy is affected due to the frequent occurrence of drug resistance [3]. Therefore, it is of great importance to reveal the potential molecular mechanism underlying drug resistance in HCC and identify novel potential effective therapeutic strategies to improve patient survival.

Recently, the emergence of a new mechanism concerning the interaction between Long non-coding RNAs (lncRNAs) and microRNAs (miRNAs) has gained widespread attention [5]. Thus, lncRNAs may function as competing endogenous RNAs (ceRNAs), sponge miRNA, subsequently influencing target mRNAs, and in some cases, miRNAs can reduce the stability of specific lncRNAs [5, 6]. A large number of studies have demonstrated that lncRNAs can interact with miRNA to mediate drug resistance. For example, UCA1 has been reported to mediate cisplatin resistance in HCC by regulating the miR-143/FOSL2-signaling pathway [7]. Moreover, the long noncoding RNA, NEAT1, has been shown to suppress the sorafenib sensitivity of HCC cells via regulating miR-335-c-Met [8]. HOX transcript antisense intergenic RNA (HOTAIR) is a 2,158-nucleotide-long lncRNA, which exists between HOXC11 and HOXC12, and regulates HOXD expression [9]. In addition, HOTAIR has been reported to be aberrantly expressed in several tumors, including cervical cancer, colorectal cancers, gastric cancer, breast cancer, and hepatic carcinoma [10–14]. However, the role of HOTAIR in the chemoresistance of HCC remains poorly understood. Therefore, we hypothesized that HOTAIR might also interact with some specific miRNAs.

In the present study, we explored the relationship between HOTAIR and sorafenib resistance and the mechanism by which HOTAIR influences the sorafenib sensitivity of HCC. Thus, our study may provide novel insight and a basis for the treatment of HCC.

## 2. Materials and Methods

### 2.1. Cell Lines and Culture

The HCC cell lines, including Huh7, Hep3B, SNU-387, and SNU-449, were obtained from the American Type Culture Collection (ATCC, Manassas, VA, USA). The Huh7 and Hep3B cell lines were maintained in high-glucose DMEM medium (Invitrogen, Carlsbad, CA, USA) supplemented with 10% fetal bovine serum (FBS, GIBCO Carlsbad, CA, USA). SNU387 and SNU-449 cells were cultured in RPMI-1640 medium (GIBCO) supplemented with 10% FBS.

### 2.2. Total RNA Extraction and Quantitative Real-Time Reverse-Transcription Polymerase Chain Reaction (qRT-PCR)

Total RNA was isolated using TRIzol reagent (Life Technologies Corporation, Carlsbad, CA, USA) according to the manufacturer's instructions. Single-stranded cDNA was synthesized using a PrimeScript RT reagent kit (Takara, Dalian, China) according to the manufacturer's procedures. The relative expression of miR-217 was examined using the Mir-X™ miRNA First-Strand Synthesis Kit (Takara). Next, qRT-PCR was performed using the SYBR Primix kit (Takara). The level of expression was calculated via the 2 − *Δ*ΔCt method and normalized to GAPDH and U6 expression, respectively.

### 2.3. Cellular Transfection

Si-HOTAIR, miR-217 mimics, the miR-217 inhibitor, and negative control were provided by GenePharma (Shanghai, China). A density of 2 × 105 Huh-7, Hep3B, SNU-387, and SNU-449 cells were plated into six-well plates and supplemented with 2 mL culture medium. The transfection of HCC cells was conducted using Lipofectamine™ 2000 (Invitrogen, Carlsbad, CA, USA) in accordance with the manufacturer's instructions. After 48 h cultivation, the cells were collected for subsequent experiments. The sequences were provided as followed:

Hsa-miR-217-5p mimics:

5'-UACUGCAUCAGGAACUGAUUGGA-3', 5'-CAAUCAGUUCCUGAUGCAGUAUU-3';

Hsa-miR-217-5p inhibitor:

5'-UCCAAUCAGUUCCUGAUGCAGUA-3';

HOTAIR-Homo-536:

Sense:5'-GCCUUCCUUAUAAGCUCGUTT-3'; Antisense:5'-ACGAGCUUAUAAGGAAGGCTT-3';

HOTAIR-Homo-1162:

Sense: 5'-CAAUAUAUCUGUUGGGCGUTT-3';

Antisense: 5'- ACGCCCAACAGAUAUAUUGTT-3';

HOTAIR-Homo-1597:

Sense: 5'- GGAAGCUCUUGAAGGUUCATT-3';

Antisense: 5'- UGAACCUUCAAGAGCUUCCTT -3';

Negative Control:

Sense:5'- UUCUCCGAACGUGUCACGUTT-3';

Antisense: 5'- ACGUGACACGUUCGGAGAATT-3';

### 2.4. Western Blot

The cells were lysed with RIPA lysate (Beyotime Biotechnology, Shanghai, China) supplemented with protease inhibitors (Roche, Basle, Switzerland). The protein concentrations were determined using a BCA Assay. The proteins (40 *μ*g) was separated by 10% SDS-PAGE and then transferred to a polyvinylidene fluoride (PVDF) membrane (Invitrogen, USA). The membranes were subsequently blocked using 5% non-fat milk in TBST for 2 h. The membranes were incubated with primary antibodies (anti-E-cadherin, ab15148, 1 : 500; anti-Vimentin, ab137321, 1/500; anti-GAPDH, ab9485, 1/500, Abcam, Cambridge, MA, USA) overnight at 4°C, and further incubated with horseradish peroxidase (HRP)-conjugated goat anti-rabbit IgG (ab205718, 1 : 2000, Abcam) at room temperature for 2 h. The proteins were visualized using ECL chemiluminescent substrate (Thermo Fisher Scientific, Inc., Waltham, MA, USA).

### 2.5. CCK-8 Assay

Cell viability was detected using a cell counting kit-8 (CCK-8) assay (Dijindo, Japan). Briefly, transfected or untransfected cells were plated into 96-well plates at a density of 5 × 10^3^ cells/well and cultured overnight at 37°C. Following treatment with a series of different sorafenib concentrations for 48 h, the cells were treated with a CCK-8 solution and incubated for 2 h at 37°C. The absorbance value at a wavelength of 490 nm was determined using a microplate reader (Bio-Tek, Winooski, VT, USA).

### 2.6. EdU Assay

A cellular proliferation assay was performed using a Click-iT EdU Imaging Kit (Invitrogen, Carlsbad, CA, USA) according to the manufacturer's instructions.

### 2.7. Immunofluorescence Assay

HCC cells were seeded onto glass slides at a density of 0.5-1 × 10^5^ cells. After 24 h of treatment, the cells were fixed with 4% paraformaldehyde for 15 min and then incubated with primary antibodies against E-cadherin (#3199, Cell signaling technology, Danvers, MA,USA) and Vimentin (#7675, CST) overnight at 4°C. The nuclei were stained with 4'6-diamidino-2-phenylindole (DAPI). Confocal fluorescence microscopy was used to observe and photograph the fluorescent sections.

### 2.8. Statistical Analysis

All quantitative values are expressed as the mean ± SD. Statistical analyses were performed using GraphPad Prism v6.0 (GraphPad Software, La Jolla, CA, USA). A Student's *t-*test was used for comparisons between two groups. A one-way analysis of variance (ANOVA) was used to analyze the statistical significance between multiple groups. A difference was considered to be statistically significant when P < 0.05.

## 3. Results

### 3.1. Sorafenib Resistance Was Increased in HCC Cells with High HOTAIR Expression

**Figure 1:**
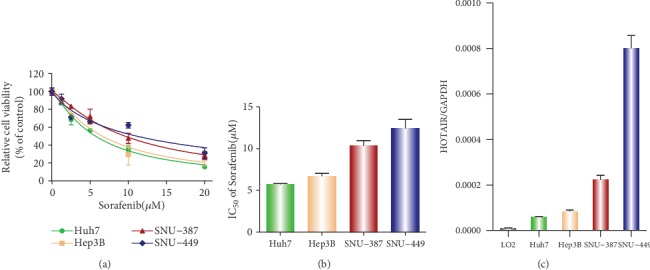
Sorafenib cytotoxicity and HOTAIR expression in hepatic carcinoma cells. (a). The sensitivity of four liver cancer cell lines to sorafenib was measured by a CCK-8 assay. (b). The IC50 of sorafenib was calculated in four liver cancer cell lines. (c). HOTAIR expression was evaluated by real-time RT-PCR in a normal liver cell line and four liver cancer cell lines.

To explore the cytotoxicity of sorafenib on HCC cells, we performed a CCK8 assay for four HCC cell lines (Huh7, Hep3B, SNU-387, and SNU-449). The cells were cultured in complete medium with different concentrations of sorafenib (0, 5, 10, 15, and 20 *μ*M) for 48 h. The results showed that the epithelial cell lines, Huh7 and Hep3B, were more sensitive than the mesenchymal cells, SNU-387 and SNU449 (Figure 1(a)). Consistent with this result, the inhibitory concentration (IC50) values of sorafenib in epithelial cells (Huh7 and Hep3B) was significantly lower compared with that of mesenchymal cells (SNU-387 and SNU449) (Figure 1(b)). Next, we used a real-time PCR assay to detect the level of HOTAIR expression. When compared to the normal hepatic cell line LO2, HOTAIR expression was significantly increased in the four cell lines (Figure 1(c)). We also found that HOTAIR expression in the Huh7 and Hep3B cells was lower than that in SNU-387 and SNU449 cells (Figure 1(c)). SNU449 cells exhibited the highest level of HOTAIR expression while Huh7 cells had the lowest.

### 3.2. Role of HOTAIR on HCC Sorafenib Sensitivity

**Figure 2:**
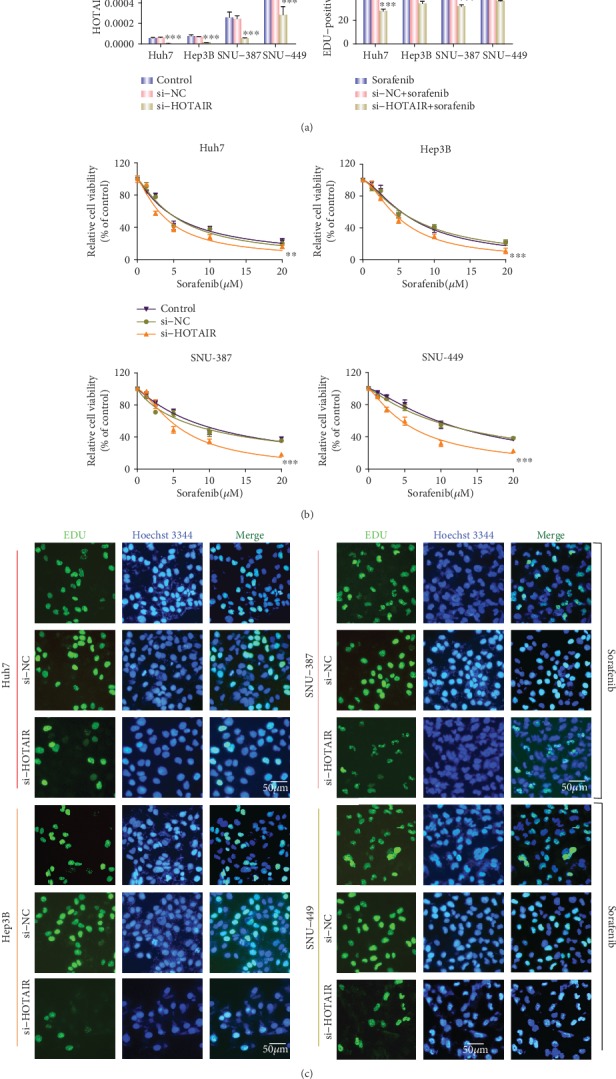
Effect of HOTAIR expression on the sensitivity of hepatic carcinoma cells against sorafenib. (a). Real-time RT-PCR analysis of HOTAIR in four HCC cell lines transfected with HOTAIR siRNA. ^∗∗∗^p < 0.001. (b). CCK8 analysis of the sorafenib chemosensitivity in four HCC cell lines transfected with HOTAIR siRNA. ^∗∗^p < 0.01, ^∗∗∗^p < 0.001. (c). The level of cellular proliferation was evaluated by an EdU assay after four HCC cell lines were transfected with HOTAIR siRNA.

Since HCC cells with higher HOTAIR expression exhibited greater resistance to sorafenib, we hypothesized that HOTAIR may be participating in HCC chemoresistance. Cellular transfection, qRT-PCR, CCK8, and EDU assays were used to test this hypothesis. The cells were transfected with si-NC or si-HOTAIR for 48 h, respectively. The efficiency of transfection was verified by qRT-PCR (Figure 2(a)). Following transfection, the cells were cultured in complete medium with different concentrations of sorafenib for 48 h. There was no difference between the control group and si-NC group regarding cell viability. However, si-HOTAIR substantially reduced cell viability in the four HCC cell lines, as observed by a CCK8 assay (Figure 2(b)). Compared to the negative control, a HOTAIR knockdown could remarkably suppress the proliferation of HCC cells in the presence of sorafenib (Figure 2(c)). These findings indicate that a HOTAIR knockdown potentiated the efficacy of sorafenib.

### 3.3. The Effect of HOTAIR on E-Cadherin and Vimentin Expression

**Figure 3:**
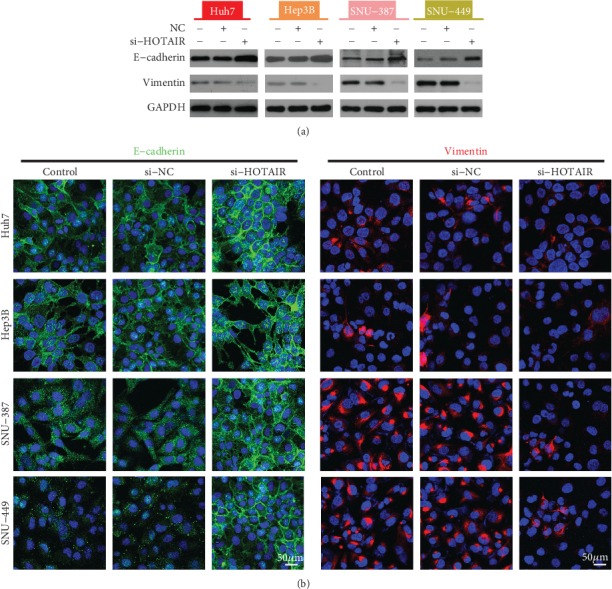
Effect of HOTAIR suppression on E-cadherin and Vimentin expression. (a). Western blot analysis for the expression of the indicated EMT markers. GAPDH protein expression was used as a protein loading control. (b). Representative immunofluorescence image of E-cadherin and Vimentin expression.

The above results showed that HOTAIR inhibition promotes the sensitivity of HCC to sorafenib. Thus, we next sought to elucidate the mechanism by which HOTAIR plays a role in sorafenib sensitivity. HCC cells transfected with si-HOTAIR were subjected to a Western blot and immunofluorescence assay. The results of western blotting showed that HOTAIR interference increased E-cadherin expression and decreased Vimentin expression compared to control (Figure 3(a)).Moreover, we found that the green fluorescence(E-cadherin) increases and the red fluorescence(Vimtein) decreases after HCC cells were transfected with si-HOTAIR(Figure 3(b)). This result was consistent with western blot, suggesting that EMT may be involved in HOTAIR-induced resistance.

### 3.4. HOTAIR Binds to miR-217 and Regulates miR-217 Expression

**Figure 4:**
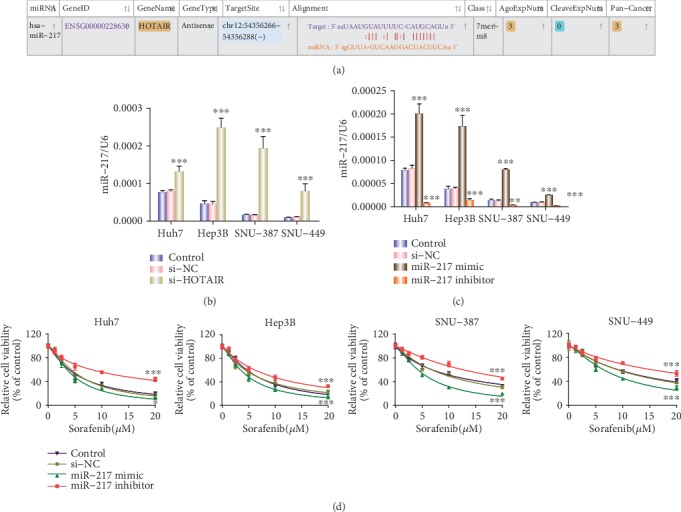
HOTAIR bound to miR-217 and regulated miR-217 expression. **(**a). Sequence alignment of the predicted HOTAIR binding sites within the mir-217 5'UTR by starbase. (b). Real-time RT-PCR analysis of miR-217 in four HCC cell lines transfected with si-HOTAIR. ^∗∗∗^p < 0.001. (c). Real-time RT-PCR analysis of miR-217 in four HCC cell lines transfected with an miR-217 mimic, inhibitor, or NC.^∗∗^p < 0.01, ^∗∗∗^p < 0.001. (d). CCK8 analysis of sorafenib chemosensitivity in four HCC cell lines transfected with an miR-217 mimic, inhibitor, or NC. ^∗^p < 0.05, ^∗∗∗^p < 0.001.

Recently, accumulating evidence has suggested that Long non-coding RNAs (lncRNAs) participant in drug resistance by interacting with miRNA [7]. To explore whether any miRNAs are involved in HOTAIR-induced resistance, a bioinformatics analysis was performed using starbase (http://starbase.sysu.edu.cn/index.php) and the results showed that miR-217 contains a HOTAIR binding site (Figure 4(a)). Next, in order to verify whether HOTAIR regulated the miR-217 expression, either si-HOTAIR or si-NC was transfected into HCC cells, and the level of miR-217 expression was detected by qRT-PCR. The results showed that miR-217 expression was significantly increased in the HCC cells transfected with si-HOTAIR (Figure 4(b)).

To test the effect of miR-217 on sorafenib sensitivity, HCC cells were transfected with miR-NC, an miR-217 mimic, or miR-217 inhibitor. The qRT-PCR results showed that treatment with the miR-217 mimic noticeably enhanced the level of miR-217 expression compared with the negative control. Inversely, inhibiting miR-217 expression with an inhibitor remarkably decreased its expression compared with the NC (Figure 4(c)). A CCK8 assay was then performed to validate the effect of miR-217 on the sorafenib sensitivity of HCC cells. As expected, miR-217 overexpression decreased cell viability, whereas an miR-217 inhibition increased cell viability compared to the negative control (Figure 4(d)).

### 3.5. Role of miR-217 on E-Cadherin and Vimentin Expression

**Figure 5:**
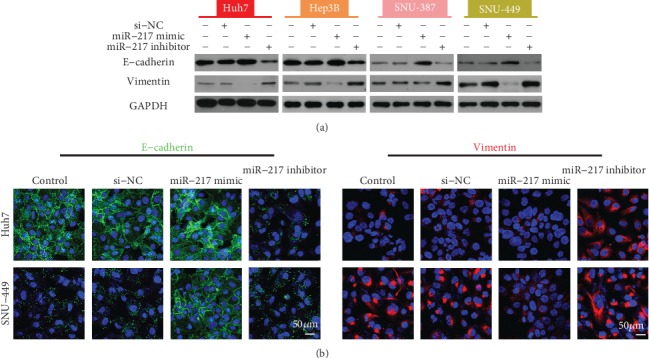
Role of miR-217 on E-cadherin and Vimentin expression. **(**a). Western blot analysis for the expression of the indicated EMT markers. GAPDH protein expression was used as a protein loading control. (b). Representative immunofluorescence image of E-cadherin and Vimentin expression.

To explore whether miR-217 could alter E-cadherin and Vimentin expression, HCC cells were transfected with miR-NC, an miR-217 mimic, or miR-217 inhibitor. The Western blot results showed that miR-217 overexpression increased E-cadherin and decreased Vimentin expression. In contrast, miR-217 inhibition had the opposite effect (Figure 5(a)). Moreover, treatment with the miR-217 mimic increased the level of E-cadherin immunofluorescence and decreased Vimentin immunofluorescence compared with the negative control. Conversely, treatment with the miR-217 inhibitor decreased the E-cadherin immunofluorescence and increased Vimentin immunofluorescence (Figure 5(b)).

### 3.6. MiR-217-Mediated Regulation of Sorafenib Sensitivity by HOTAIR Knockdown

**Figure 6:**
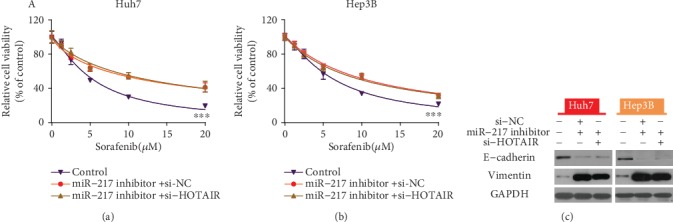
MiR-217 mediated the regulation of sorafenib sensitivity by HOTAIR knockdown. A-B. The results of the CCK-8 assay showed that there was no difference in cell viability between cells treated with an miR-217 inhibitor or an miR-217 inhibitor + si-HOTAIR. ^∗∗∗^p < 0.001.C. The results of the Western blot assay showed that there was no difference in E-cadherin and Vimentin expression between the cells treated with the miR-217 inhibitor and the miR-217 inhibitor + si-HOTAIR.

To investigate miR-217-mediated modulation of HOTAIR-mediated sensitivity in HCC cells, we transfected Huh7 and Hep3B cells with miR-217 inhibitor, and subsequently transfected them with si-NC or si-HOTAIR. The results of the CCK-8 assay showed that there was no difference in cell viability between treatment with the miR-217 inhibitor and miR-217 inhibitor + si-HOTAIR (Figure 6(a)-6(b)). The results of the Western blot showed that there was no difference in E-cadherin or Vimentin expression between the cells treated with an miR-217 inhibitor and those treated with the miR-217 inhibitor + si-HOTAIR (Figure 6(c)).

## 4. Discussion

Sorafenib has been widely used as first-line chemotherapy for advanced HCC; however, some patients eventually acquire sorafenib resistance and experience tumor recurrence and metastasis. Therefore, it is important to avoid sorafenib resistance in the clinical treatment of HCC. Recently, lncRNAs have been found to interact with miRNA and participate in drug resistance [7, 8]. Thus, the role of lncRNA in HCC has attracted increased attention.

HOTAIR has been shown to function as an oncogene since it is overexpressed in numerous cancers and is involved in physiological and pathological progression, including cellular proliferation, apoptosis, angiogenesis, invasion, and metastasis [15, 16]. Moreover, Yang et al. reported that HOTAIR overexpression predicts tumor recurrence in HCC patients [17]. HOTAIR is overexpressed and regulates PTEN methylation in laryngeal squamous cell carcinoma [18]. In addition, emerging evidence has demonstrated that HOTAIR expression is involved in drug resistance and HOTAIR overexpression has been shown to promote chemoresistance in various malignancies [19, 20]. In the present study, we found that HCC cells with higher HOTAIR expression exhibited greater resistance to sorafenib, indicating that HOTAIR may be a pro-oncogenic factor. In addition, a HOTAIR knockdown inhibited the proliferation of HCC cells in the presence of sorafenib.

The epithelial-mesenchymal transition (EMT) process is characterized by reduced epithelial characteristics and increased mesenchymal properties. HOTAIR has been reported to promote tumor cell invasion and metastasis by regulating the EMT [21, 22]. Moreover, previous studies have revealed that the EMT is one of several potential mechanisms involved in the acquired resistance to sorafenib in HCC treatment [3]. Therefore, we sought to elucidate whether EMT is associated with the sorafenib sensitivity induced by HOTAIR inhibition. By comparing the changes in the expression of EMT makers before and after interference, we found that a HOTAIR knockdown could increase E-cadherin and decrease Vimentin expression, indicating that a HOTAIR knockdown inhibits the EMT process.

Previous research has found that HOTAIR negatively regulates the expression of miRNAs primarily by functioning as a competing endogenous RNA (ceRNA) sponge (e.g., miR-34a and miR-218) to contribute to drug resistance in both gastric and colorectal cancer, respectively [23, 24]. In the present study, we used starbase to predict and found that miR-217 contains a binding site for HOTAIR. Moreover, HOTAIR could negatively regulate the expression of miR-217. Treatment with a miR-217 mimic could enhance sorafenib sensitivity and E-cadherin expression and reduce Vimentin expression. In contrast, treatment with the miR-217 inhibitor had the opposite effect, promoting sorafenib resistance and the EMT. Finally, compared with the mir-217 inhibitor alone and the HOTAIR siRNA + mir-217 inhibitor, there was no significant difference in cell viability, indicating that mir-217 mediates HOTAIR-mediated sorafenib sensitivity.

In conclusion, our study has shown that a knockdown of the lncRNA, HOTAIR, sensitizes HCC cells to sorafenib via inhibiting the EMT. More importantly, a knockdown of lncRNA HOTAIR leads to an upregulation in mir-217 expression and elevated sorafenib sensitivity to HCC, which may provide a more effective treatment for patients with HCC.

## Data Availability

The data used to support the findings of this study are included within the article.
